# The characteristics of CD4^+^T-helper cell subset differentiation in experimental *Clonorchis sinensis*-infected FVB mice

**DOI:** 10.22038/ijbms.2020.39436.9350

**Published:** 2020-12

**Authors:** Delong Kong, Xiangyang Li, Beibei Zhang, Chao Yan, Renxian Tang, Kuiyang Zheng

**Affiliations:** 1 Jiangsu Key Laboratory of Immunity and Metabolism, Department of Pathogenic Biology and Immunology, Laboratory of Infection and Immunity, Xuzhou Medical University, Xuzhou, 221004, Jiangsu Province, People’s Republic of China

**Keywords:** Adaptive immunity, Clonorchiasis, Inflammation, Treg cells, Th17 cells

## Abstract

**Objective(s):**

Immune responses are tightly regulated in the development of clonorchiasis. However, the adaptive immune regulatory pathway contributing to the pathological processes of *clonorchis sinensis (C. sinensis)* infection is still not clear. In this study, we assessed the dynamic changes of CD4^+^T cell subsets and the related cytokines as well as transcription factors during *C. sinensis* infection.

**Materials and Methods:**

We used female FVB mice to establish the infection model. H&E and Masson’s stain were performed in 2 or 8 weeks post-infection (PI) liver of *C. sinensis*. The percentages of splenic Th1, Th2, and Treg in CD4+T cells were detected by flow cytometry. The transcription factors T-bet, GATA3, Foxp3, and RORγt gene expression were detected by qPCR. The protein expression of IL-10, IL-17, IL-4, IL-2, and Tumor Necrosis Factor-α (TNF-α) were examined using ELISA.

**Results:**

The percentages of splenic Th1, Th2, and Treg in CD4^+^T cells were significantly increased in both 2 and 8 weeks PI of *C. sinensis*, while the ratio of Treg/Th17 as well as the percentage of Treg in serum was gradually increased during the development of infection. The expressions of T-bet, GATA3, Foxp3, and RORγt were increased in 8 weeks PI. Serum levels of IL-10, IL-17, IL-4, and IL-2 were profoundly increased in infected mice, while the concentrations of TNF-α increased to peak two weeks PI.

**Conclusion:**

Our results suggested that the imbalance of CD4^+^T cell subsets may regulate and contribute to an appropriate compromise between pathology, tissue repair, and elimination in a susceptible murine host.

## Introduction

Clonorchiasis is a neglected food-borne parasitic disease, which is caused by *Clonorchis sinensis *(*C. sinensis*), a kind of helminth parasite affecting appoximately 30 million people in Eastern Asian including China, Korea, Japan, and Vietnam (1). Human or other mammals such as dogs and cats can get infected by ingestion of raw or undercooked fresh-fish that contains metacercariae of *C. sinensis,* and the worms become mature in the intrahepatic bile ducts, which lead to obstruction, inflammation, and fibrosis of bile ducts, as well as blockage of bile transport. Importantly, *C. sinensis* infection can potentially result in cholangiocarcinoma and are recently considered as a type of biological carcinogen for humans. 

The mammalian immune response against helminths consists of the type 2 phenotype characterized by IgE antibody production, eosinophilia, mastocytosis, and

specific forms of fibrotic wound repair under the control of many types of cytokines (2). Although parasites require co-stimulation to induce optimal type 2 phenotype immune response, they mostly lack the microbes-associated molecular patterns (MAMPs) that are important stimulants to induce antigen-presenting cell activation, co-stimulatory molecule expression, and the secretion of cytokines (3). Moreover, the ongoing refinement of our understanding of type 2 immune response and the recent description of new T-helper cell subsets indicated that it was necessary to further study the adaptive immune responses in the context of helminth infection. CD4^+^T helper (Th) cells play important roles in orchestrating the host’s immune responses against the foreign antigen by differentiating into functionally distinct effector Th cell subsets including Th17 and regulatory T cells (Tregs) as well as conventional Th1 and Th2 subsets (4). Different Th cells are characterized by the production of distinct cytokines and play different roles in a variety of diseases. For example, Th1 cells are critical for elimination of intracellular microbes, while Th2 cells secreting IL-4, IL-5, and IL-13 play an important role in host defenses against extracellular pathogens. IL-17-secreting Th17 is important for protective immune responses to extracellular pathogens, such as *Klebsiella pneumonia* (5), *Porphyromonas gingivalis* (6), and *Bacteroides fragilis* (7). In addition, several studies showed that IL-17 and Th17 were also responsible for severe immunopathology in some parasitic diseases such as toxoplasmosis (8) and leishmaniasis (9). CD4^+^CD25^+^Foxp3^+ ^regulatory T (Treg) cells belong to suppressive subsets that can negatively regulate immune responses by production of anti-inflammatory cytokines IL-10 and TGF-β or by cell-cell contact-dependent immune suppression. Several experimental pieces of evidence suggested that Treg cells could improve parasite survival and regulate worm-induced pathology (10-12). 

It has been reported that increased production of Th2-associated anti-inflammatory cytokines such as IL-4, IL-5, and IL-13 was found in FVB mice infected by *C. sinensis* (13). Our previous study demonstrated that hepatic Th2 and Treg cell subsets were positively correlated with hepatic pathological damage in the development of clonorchiasis (14). However, there is still very little *in vivo* data available to show the cross-regulation between Th17, Th1, Th2, and Treg cell differentiation during *C. sinensis* infection. Therefore, to further assess the alteration of Th cell repertories during *C. sinensis* infection, the present study evaluated cytokine production and cell population of Th1, Th2, Th17, and Treg cells in different infection stages, together with their related transcription factor expression in *C. sinensis* infected mice.

## Materials and Methods

***Mice and infection***

Eight-week-old female FVB mice were purchased from the Model Animal Research Center of Shanghai (Shanghai China). They were bred under specific pathogen-free conditions. All animal experiments complied with the Guide for the Care and Use of Laboratory Animals of the Ministry of Health of China and were approved by the Animal Ethics Committee of Xuzhou Medical College (No. SCXK<SU>2010-0003). Mice were infected with thirty *C. sinensis metacercariaes* in 100 μl of 0.9% NaCl orally, which were isolated from infected *Pseudorasbora parva* purchased from Guangzhou in China. 

***Histological analysis***

Livers were removed at the indicated time points and fixed in 10% buffered formalin. The paraffin-embedded section (3.5 μm) was stained with H&E and Masson’s trichrome stain. The sections were examined with an Olympus microscope.

***Flow cytometric analysis***

At the indicated time points, we first collected circulating blood from the mice which were then executed with anesthesia; liver, and spleen tissues were isolated for later research. The single-cell suspensions were prepared from spleens packed in sterile nylon in PBS and then red blood cells were dissolved by ACK lysing buffer. To purify CD4^+^T cells, anti-mouse-CD4 Micro-beads (MACS, Miltenyi Biotec) were used according to the manufacturer’s instructions. For Th1, Th2, and Th17 subset examination, approximately 1×10^6^ cells were incubated with 20 ng/ml phorbol 12-myristate-13-acetate (PMA) and 1 μg/ml ionomycin in the presence of 2 mmol/ml monensin (Sigma-Aldrich, USA) for 4 hr (37 °C, 5% CO_2_). For surface staining, the cells were incubated with the following fluorescently labeled monoclonal antibodies for 30 min at room temperature in the dark: CD4-PE-Cy5 (eBiosciences, CA, USA). Subsequently, they were permeabilized in paraformaldehyde and Perm/Fix solution according to the manufacturer’s instructions; then cells were stained intracellularly with anti-mouse FITC-IFN-γ, APC-IL-4, and PE-IL-17 (eBiosciences, CA, USA). To examine Treg cells, firstly, made cell suspension stain with PE-cy5-anti-CD4 and FITC-anti-CD25 at room temperature for 30 min, treated with eBioscience fix/perm mixture, and incubated with anti-mouse Foxp3 antibodies according to the manufacturer’s instructions (eBiosciences, CA, USA). All of the stained cells were detected using flow cytometry (FACS Calibur), and the data were analyzed using the cellquest software package (BD Biosciences, CA, USA).

***Cytokine analysis***

The release of TNF-α, IL-4, IL-2, IL-17, and IL-10 in serum was measured using commercially available ELISA kits according to the manufacturers’ instructions (eBioscience, CA, USA).

***RNA analysis***

Liver sections were crushed totally in liquid nitrogen and then lysed on ice for 30 min. Hepatic total RNA was extracted with TRIzol reagent (Tiangen Inc., Beijing, China). cDNA synthesis was performed using the A commercial TIANScript RT kit® (Tiangen Inc., Beijing, China) according to the manufacturer’s instructions. Real-time polymerase chain reaction (RT-PCR) was performed in 20 μl which contained 10 μl LightCycler FastStart DNA Master ((Roche Applied Science, Mannheim, Germany), 1 μl cDNA, 7 μl RNase/DNase-free water, and 500 nM of each primer. The following primer sequences were listed in Table 1. Quantification was determined by the standard curve and 2-^ΔΔCt^ methods. The β-actin was used as an internal control to normalize all PCR products.

***Statistical analysis***

Data obtained from different groups were analyzed using SPSS (Release 16.0 standard version, SPSS Inc., Chicago, IL, USA) and the GraphPad software package (Graphpad, San Diego, CA, USA). All data were shown as mean±standard error (SE), correlations were calculated using Pearson’s correlation and the data were considered statistically significant when *P*-values were≤0.05 by a two-tailed t-test. 

**Table 1:** The primer pairs were used in this study

Name		Sequence（**5′→3′**）
β-actin	Forward primer	CGTGGGCCGCCCTAGGCACCA
	Reverse primer	TTGGCCTTAGGGTTCAGGGGGG
Foxp3	Forward primer	GAGAAAGCGGATACCAAA
	Reverse primer	TGTGAGGACTACCGAGCC
RORγt	Forward primer	TGCAAGACTCATCGACAAGG
	Reverse primer	AGGGGATTCAACATCAGTGC
GATA-3	Forward primer	GGCACGATCCAGCACAGAAG
	Reverse primer	TTTATGGTAGAGTCCGCAGGC
T-bet	Forward primer	TCCCATTCCTGTCCTTCACCG
	Reverse primer	CTTTCCACACTGCACCCACTTG

**Figure 1:**
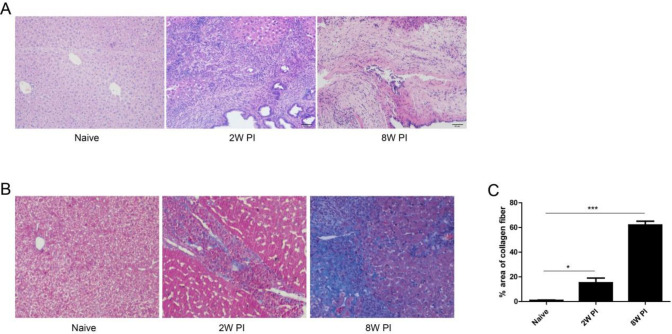
Representative hepatic histology images from naive or *Clonorchis sinensis-*infected FVB mice 2 weeks and 8 weeks post-infection (PI). (A) Liver tissues were stained with H&E. (B) Collagen depositions in the liver of *Clonorchis sinensis*-infected mice were detected by the method of Masson’s trichrome staining (collagen fibers were stained blue) and quantitative analysis of positive stain of collagen fiber (C). Data were analyzed and presented as the mean±SEM. **P<0.01, ***P<0.001, compared with the Naïve group

**Figure 2:**
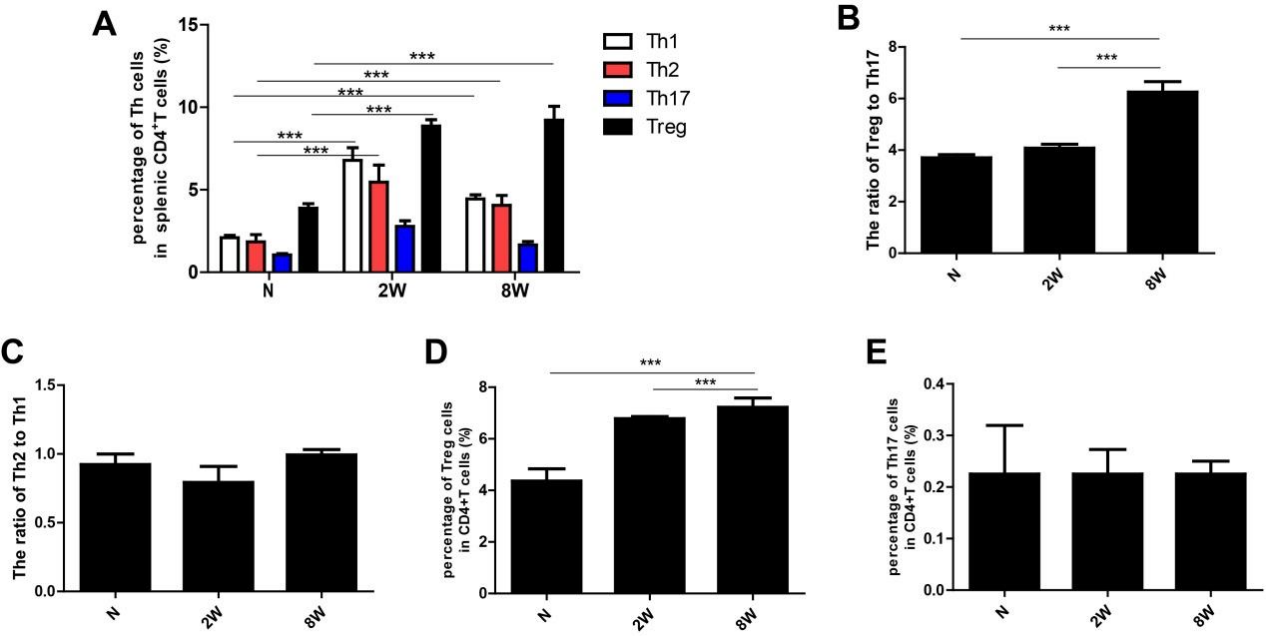
Liver sections from *Clonorchis sinensis-*infected and non-infected mice were homogenized, and then Th1, Th2, Th17, and Treg cell percentages among CD4^+^T cells were analyzed by flow cytometry. (A) Statistical analysis of the dynamic expression of diverse CD4^+^T cell subsets of liver tissues in *clonorchis sinensis* infected and non-infected mice. The Treg/Th17 ratio (B) and the Th2/Th1 ratio (C) were calculated. Quantitative changes of Treg (D) and Th17 (E) percentages in serum CD4^+^T cells. Data are expressed as mean±SEM. ***P<0.001

**Figure 3:**
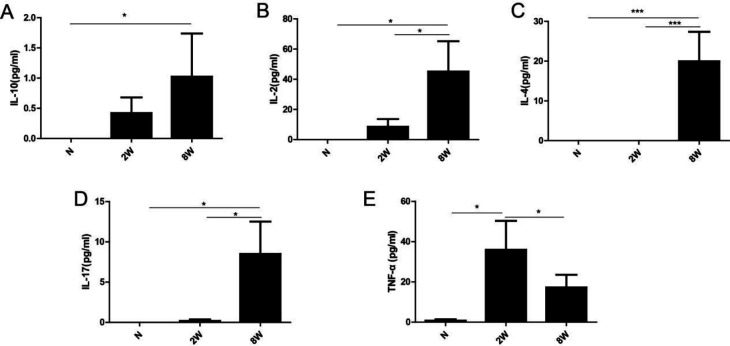
Dynamic changes of IL-10, IL-17, IL-4, IL-2, and TNF-α cytokines in acute and chronic stages of *Clonorchis sinensis* infected mice detected by the method of enzyme-linked immunosorbent assays (ELISA). Data are expressed as mean±SEM. *P<0.05, **P<0.01, and ***P<0.001

**Figure 4:**
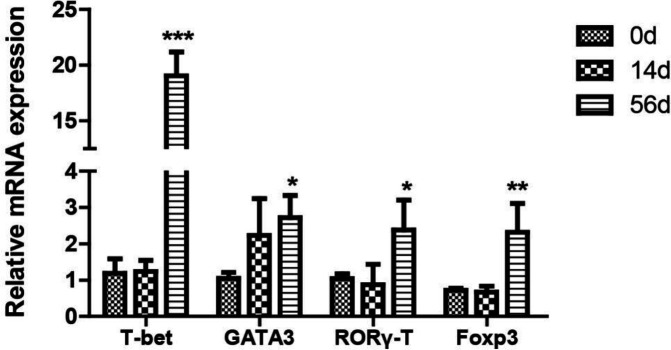
The expressions of T-bet, GATA3, Foxp3, and RORγt in liver tissues from FVB mice infected with *clonorchis sinensis* at 0 weeks, 2 weeks, and 8 weeks detected by qPCR. Data are expressed as mean±SEM. *P<0.05, **P<0.01, and ***P<0.001

## Results

***Liver histopathological changes in mice model of C. sinensis infection***

To evaluate the pathological characteristics in infected liver tissues, the liver samples of non-infected mice and 2 or 8 weeks of PI with* C. sinensis* were harvested. H&E and Masson stain were performed. Our results showed that a number of inflammatory cells appeared in the liver tissues 2 weeks PI. With the prolongation of the infection period, intrahepatic bile duct epithelia were proliferated in the liver tissue 8 weeks PI and the hepatic lobule structure was destroyed seriously accompanied by the necrolysis of hepatic cells (Figure 1A). Furthermore, a large area of fibrogenesis even emerged in the portal and periportal areas during the development of infection (Figures 1B-C).

***Alteration of CD4*** ^+^ ***T cell subsets and the ratio of Treg /Th17 as well as Th1/Th2 ratio during C. sinensis infection***

The percentages of splenic Th1, Th2, and Treg were significantly increased in both 2 and 8 weeks PI of* C. sinensis*-infected mice, compared with non-infected FVB mice. However, there was no statistical difference in the Th17 subset in different groups (Figure 2A). The ratio of Treg/Th17 was gradually increased during the development of infection. Moreover, the Treg/Th17 ratio was significantly higher 8 weeks PI compared with 2 weeks PI of* C. sinensis*-infected mice (Figure 2B). Although there were no statistical differences in the ratio of Th2/Th1 between 2 weeks PI and 8 weeks PI, it had a similar tendency as the Treg/Th17 ratio (Figure 2C). We also detected the expression of Th17 and Treg percentages in serum, the results showed the percentage of Treg was gradually increased from the acute infection stage (2 weeks PI) to the chronic stage (8 weeks PI) (Figure 2D), while there were no statistical differences in Th17 percentage between *C. sinensis*-infected mice and control mice (Figure 2E).

***Change of CD4*** ^+^ ***T cell subsets-related cytokines in C. sinensis infected mice***

To further ascertain the molecular response during the infection of *C. sinensis*, we measured the CD4^+^T cell subsets-related cytokines, including IL-10, IL-17, IL-4, IL-2, and TNF-α by ELISA methods. As demonstrated in Figure 3A, IL-10 concentration markedly increased in 8 weeks PI mice compared with non-infected mice. The expressions of IL-2, IL-4, and IL-17 were markedly increased in chronic stages of *C. sinensis* infected mice compared with non-infected mice and 2 weeks PI mice (Figure 3B-D). However, the concentration of TNF-α in 2 weeks PI mice was significantly higher than in the chronic stage (Figure 3E). 

***CD4*** ^+^ ***T cell subsets-related transcription factors expression in liver tissues of C. sinensis infected mice***

To further investigate the change of CD4^+^T cell subsets-related transcription factors in *C. sinensis*-infected mice, we measured their specific master regulators T-bet (Th1), GATA3 (Th2), Foxp3 (Treg), and RORγt (Th17) by qPCR. The results showed that the expressions of those transcription factors were significantly higher 8 weeks PI compared with the non-infected group (Figure 4).

## Discussion

Parasites represent a diverse group of pathogens that often trigger immune responses during the development of infections(15). James F P McConnell, a professor of pathology firs reported that *C. sinensis* lived in human bile ducts from a post-mortem examination of a 20-year-old Chinese man-undertaken at the Medical College Hospital in Calcutta, India (1). Although there is a sound understanding that the histopathology of clonorchiasis is mainly characterized by hyperplasia of the intrahepatic bile-duct epithelium, followed by periductal and liver fibrosis, the immune regulatory pathway contributing to the pathological processes is still not clear (16). Recently, the immunological role of functional T-cell subsets in pathogen infections such as fungal, viral, and parasitic infections has become a research focus. A number of studies demonstrated that the relative balance of Th1 and Th2 immune response is a key mediator for regulating the pathological process of infectious disease-induced liver injury. The Th2-related chemokines and cytokines have been identified to play a dominant role in the induction of Th2 cell activation and decrease the worm burdens as well as disease severity in helminthic infections (17, 18). For example, after infection with *F. hepatica*, the immune response is proinflammatory Th-1 type lasting for 4–6 weeks. This immune response switched while the adult flukes began to enter the bile duct to the Th2 immune response (19, 20). However, studies about Schistosoma infection found that both Th1 and Th2 responses were associated with protection (21, 22). 

Th1, Th2, Th17, and Treg cells are the four major subpopulations consisting of CD4^+^T cells according to differentiation and different biological functions. Recent studies have demonstrated that the immune imbalance between Th1/Th2 and Th17/Treg may be the most direct and most important factor in the pathogenesis of diseases (23). There is increasing evidence that toll-like receptors –a class of pattern recognition receptors– induce innate immune responses and orchestrate the adaptive immune responses including the development and differentiation of Th cells (24). In our previous study, we showed dramatic changes of TLR2 and TLR4, and production of IFN-γ, IL-6, TNF-α, and IL-10 in the spleen were regulated by TLR4 as demonstrated in a murine model of clonorchiasis using TLR4 defective mice (25). *In vitro* study also showed that TLR4 modulated the maturation of bone marrow-derived dendritic cells (BMDCs), which may affect the polarity of Th cells, and induced a Th2/Treg bias during C. sinensis infection (26). 

The differentiation of T cells into diverse subsets requires the expression of different transcription factors. The transcription factor T-bet not only controlled the production of the characteristic cytokine IFN-γ of Th1 cells but also inhibited the secretion of the Th2 type cytokines such as IL-4. Meanwhile, IFN-γ also could promote the expression of T-bet, which forms a complex regulatory network of immune responses during helminth infection. Furthermore, IL-4 as well as IL-13 could potently induce liver fibrosis which inhibited liver injuries due to accumulated Th1 like responses (such as TNF-α and IFN-γ) at the early stages of helminthic infection. The pathogenic cytokine IL-17 could be produced by Th17 cells, where the transcription factor RORγt would show a significant expression(27, 28). Treg cells capable of suppressing parasite-specific Th1, Th2, and Th17 responses induced the immune balance, and immunopathology control, to a certain extent, is mediated by producing IL-10 and TGF-β (29, 30), Study also showed that Treg could promote the survival of worms and facilitate chronic infection by suppressive Th1 or Th17 immune reaction (31-33). In the present study, a skewed ratio of Treg/Th17 in FVB mice was found after infection, which suggested that an imbalance between Treg and Th17 might be associated with survival of *C. sinensis* and liver fibrosis caused by *C. sinensis* during the infection course. Interestingly, a study about atopic dermatitis showed the expansion of thymus-derived regulatory T cells exhibiting a Th2-like phenotype which aggravated the skin inflammation(34). In our present study, it is still not clear whether the massive differentiation of Treg cells can also secrete Th2 cytokines to promote inflammatory response and thus resist the invasion of worms, which is worthy to be further studied.

## Conlusion

Our present study showed a potential role of imbalance in Treg/Th17, Th1/Th2, as well as their related molecules in the pathogenic process of clonorchiasis, which may lead to an appropriate compromise between pathology, tissue repair, and elimination. Further studies should be warranted to determine the mechanism for the disturbed balance of Th1, Th2, Th17, and Treg during* C. sinensis* infection, which in turn may favor the development of therapeutic strategies.
